# Subcortical Brain Abnormalities and Clinical Relevance in Patients With Hemifacial Spasm

**DOI:** 10.3389/fneur.2019.01383

**Published:** 2020-01-15

**Authors:** Hui Xu, Chenguang Guo, Feifei Luo, Romina Sotoodeh, Ming Zhang, Yuan Wang

**Affiliations:** ^1^Department of Medical Imaging, The First Affiliated Hospital of Xi'an Jiaotong University, Xi'an, China; ^2^Key Laboratory of Biomedical Information Engineering of Education Ministry, Institute of Biomedical Engineering, Xi'an Jiaotong University, Xi'an, China; ^3^Faculty of Dentistry, McGill University, Montreal, QC, Canada

**Keywords:** hemifacial spasm, orofacial muscle contractions, subcortical brain abnormalities, affective symptoms, translational medicine

## Abstract

**Purpose:** Hemifacial spasm (HFS), a rare neuromuscular movement disorder, is characterized by unilateral, irregular, and paroxysmal facial muscle contractions. To explore the central neural mechanisms of HFS, we conducted vertex-wise shape analyses to investigate volume and shape alterations of subcortical structures, which could help to better understand the abnormality in distinct subcortical regions and determine alternative biomarkers of HFS.

**Methods:** Thirty patients with HFS and 30 age- and sex-matched healthy controls provided written informed consent. T1-weighted structural magnetic resonance imaging (MRI) data were collected from all participants. Vertex-wise shape analyses were performed to assess the volume and shape alterations of subcortical structures following HFS. *Post hoc* correlations with spasm severity and measures of mood dysfunction were applied to characterize subcortical brain alterations.

**Results:** Compared with healthy controls, patients with HFS showed increased volume in the right caudate specifically. Furthermore, patients exhibited significant shape atrophy in the anterior medial aspect of left pallidum, together with shape expansion in the anterior ventrolateral aspect of right caudate head. In addition, shape alteration in right caudate was positively correlated with both anxiety and depression severity in patients with HFS.

**Conclusions:** This is the first study to employ vertex-wise shape analysis to investigate subcortical brain abnormalities in patients with HFS. Our findings provide compelling evidence for subcortical brain alterations specific to HFS, and further may shed light on the pathophysiology of HFS and apply to the translational medicine.

## Introduction

Hemifacial spasm (HFS), a rare neuromuscular movement disorder, is characterized by unilateral, irregular, and paroxysmal facial muscle contractions (1, 2). Its spasms usually start in the orbicularis oculi muscle of the eyelid (3). As this disorder progresses, spasms spread to the orbicularis oris around lips and buccinator muscles at the cheekbone (4). HFS is not a life threating condition with low incidence in the general population (5), but it is more prevalent among older adults and inevitably causes social embarrassment and mental distress, which can affect patients' functioning and quality of life (6). Notably, patients with HFS demonstrate negative emotional symptoms, which have been reported to be related to brain structural alterations (7).

Subcortical structures including the basal ganglia and parts of the limbic system (8) have vital roles in learning, attention, memory, motor control, as well as emotion (9). Furthermore, they are integrally involved in cognitive executive functions through their structural and functional connectivity with high-order cortical areas (10). Prior studies have revealed alterations of subcortical regions in various psychiatric disorders including autism spectrum disorders (11), depression (12), and schizophrenia (13). However, little is known about the contribution of subcortical abnormalities to the progress of HFS, and it is not clear whether those alterations cause or result from HFS symptoms. To date, only one study has observed subcortical alterations in HFS, which demonstrated reduced gray matter volume in the thalamus, putamen, pallidum, and amygdala in patients with HFS compared to healthy volunteers (7). The data were processed by voxel-based morphometry (VBM) and traditional volumetric (i.e., total volume) approach with inherent limitations. Furthermore, the findings reflect a mix of variations in shape, volume, and position of subcortical structures, and depend on smoothing extents and accurate classification of tissue type.

Vertex-wise shape analysis has been applied in studies of subcortical morphology in various disorders (14–16), and has been approved to overcome the limitations from the VBM and traditional volumetric approach. This method uses a joint shape and appearance model to robustly determine the subcortical boundary. It then provides a local and direct measure of geometric change that does not depend on the tissue-type classification or arbitrary smoothing extent (17). To date, vertex-wise shape analysis has not been performed to characterize subcortical brain abnormalities in HFS condition.

Hence, in current study, we conducted vertex-wise shape analyses to investigate volume and shape alterations of subcortical structures in patients with HFS compared with matched healthy controls. The objectives of this study are to: (1) identify subcortical volume alterations in patients with HFS, (2) identify subcortical shape abnormalities in patients with HFS. Finally, we will test whether these subcortical abnormalities are associated with clinical data.

## Materials and Methods

### Participants

Thirty patients with HFS (18 females, mean age 48.80 ± 11.73 years) were recruited from neurological clinics. The diagnosis of HFS was assessed by two experienced neurologists according to the following criteria as typical hemifacial muscle spasms with involuntary and intermittent onset and no neurological deficit or sensory loss. Exclusion criteria were: secondary HFS caused by tumors and cysts, significant premorbid psychiatric or neurological history, alcohol or substance misuse, and MRI contra-indicators (e.g., metal implants, claustrophobia). Thirty age- and sex-matched healthy controls were recruited (18 females, mean age 49.77 ± 11.61 years). Participants had no history of psychiatric or neurological illness, alcohol or substance misuse. This study was conducted in accordance with the Declaration of Helsinki and had full ethical approval from the ethics committee of the first affiliated hospital of Xi'an Jiaotong University. Each participant gave written informed consent.

### Neuropsychological Assessment

All subjects underwent a structured clinical interview and completed a brief psychological assessment. The Hamilton Anxiety Rating Scale (HAM-A) was used to evaluate anxiety symptoms (18, 19), and the Hamilton Depression Rating Scale (HAM-D) measured feelings of depression (20). The obtained data were reviewed by a psychiatrist who was blinded to the experimental groups. In addition, patients with HFS were also assessed by Cohen evaluation scale (21) to quantify severity of facial muscle contraction [0−4 scale: 0 = none; 1 = increased blinking caused by external stimuli; 2 = mild, noticeable fluttering, not incapacitating; 3 = moderate, very noticeable spasm, mildly incapacitating; 4 = severely incapacitating (unable to drive, read, etc.)].

### MRI Data Acquisition

Neuroimaging data from patients with HFS and healthy controls were acquired on a 3.0-T scanner (Signa HDxt; GE Medical Systems, Waukesha, WI, USA) with an 8-channel phased-array head coil. For each subject, a T1-weighted high-resolution structural image was acquired using axial fast spoiled gradient recalled sequence with the following parameters: field of view (FOV) = 256 mm × 256 mm, matrix size = 256 × 256, time of repetition (TR) = 10.7 ms, time of echo (TE) = 4.9 ms, flip angle (FA) = 15°, voxel size = 1.00 × 1.00 × 1.0 mm^3^, and scan duration = 4 min and 51 s. Next, a resting-state functional magnetic resonance image scan and a diffusion tensor image scan were carried out immediately after the survey, but they were not discussed in this study.

### Statistical Analysis of Demographical and Clinical Variables

Demographical and clinical characteristics of all subjects were analyzed using SPSS 25.0 software (Statistical Package for Social Sciences, Release 25.0, IBM, Chicago, IL), and parametric and non-parametric statistics were used as appropriate. Group differences in age, HAM-A score, HAM-D score were evaluated using independent samples *t*-tests; chi-square test was used to estimate sex difference between groups.

### Vertex-Wise Shape Analyses

MRI data analysis was processed using FSL tools (FMRIB Software Library v6.0, https://fsl.fmrib.ox.ac.uk/fsl/) (22). First, brain tissue volume, normalized for participant head size, was estimated with SIENAX (Structural Image Evaluation, using Normalization, of Atrophy) (23), part of FSL. By using SINEAX, the volumes of neocortical gray matter (GM), total GM, white matter (WM), cerebral spinal fluid (CSF), total intracranial volume (TIV), and a volumetric scaling factor were acquired. Second, FSL-integrated registration and segmentation toolbox (FIRST, part of FSL, https://fsl.fmrib.ox.ac.uk/fsl/fslwiki/FIRST) (17), a model based automated registration and segmentation tool, was used to specifically investigate neuroanatomical alterations of subcortical structures in shape. Briefly, all subcortical structures were segmented, from high-resolution structural images, based on shape and appearance models using Gaussian assumptions combined with a Bayesian probabilistic approach (17). Then, the volumetric labels in terms of meshes were automatically parameterized using deformable surfaces of each subcortical structure. And the normalized intensities along the surface of meshes were sampled and modeled. Finally, a shape appearance model was performed based on multivariate Gaussian assumptions. The shape was expressed as a mean with modes of variation. Quality of segmentation and registration of all subcortical structures were manually checked and confirmed for each subject.

For the normalized volume of brain tissue (GM, total GM, WM, CSF, and TIV) from SIENAX, we conducted a univariate mixed ANOVA with normalized volume as a dependent variable, group (patients with HFS and healthy controls) as a between-subjects factor and brain tissue as a within-subjects factor. SPSS 25.0 software was used for this statistical analysis.

Vertex-wise shape analyses of subcortical structures were performed using a general linear model (GLM) with permutation-based non-parametric testing (5,000 times permutation using randomize) to examine group differences. Maps showing significant group differences between patients with HFS and healthy controls were generated by thresholding the images of t statistics with cluster-based family-wise error (FWE) correction of *P* < 0.05.

### Quality Control of Structural MRI

For anatomical data, we checked the image quality to ensure that there was no apparent motion artifact in each subject. All scans were field inhomogeneity corrected. During the structural MRI analysis, we inspected any artifact that could affect the processing, including segmentation, normalization, etc. All structural MR images from each participant were good for further analysis, so we included all participants (30 patients with HFS and 30 healthy controls, details seen in Table 1) in this study.

**Table 1:** Demographics and clinical variables of patients with HFS and healthy controls.

	**Patients with HFS (SD)**	**Healthy controls (SD)**	**Significance, *P*-value**
Age (years)	48.80 (11.73)	49.77 (11.61)	*t*_58_ = 0.32, *P* = 0.75
Sex (female/male)	18/12	18/12	χ^2^ <0.01, *P* > 0.99
Duration of disease (years)	3.38 (3.53)	NA	NA
Scores of Cohen	2.93 (0.74)	NA	NA
Scores of HAM-A	5.23 (2.66)	0.23 (0.57)	*t*_58_ = 10.06, *P* < 0.01^*^
Scores of HAM-D	4.97 (2.77)	0.23 (0.77)	*t*_58_ = 9.01, *P* < 0.01^*^

*HFS, hemifacial spasm; SD, Standard Deviation; Scores of Cohen, spasm severity rating via the Cohen evaluation scale; HAM-A, Hamilton Anxiety Rating Scale; HAM-D, Hamilton Depression Rating Scale; NA, not applicable*.

* *Significant difference between patient and control groups*.

### Volumetric Analyses

The absolute volumes of subcortical structures were calculated from FIRST using *fslstats* command (https://fsl.fmrib.ox.ac.uk/fsl/fslwiki/Fslutils). Then, normalized volumes of subcortical structures were obtained by multiplying those absolute volumes by a volumetric scaling factor of each subject from SINEAX.

To investigate volumetric alterations of subcortical structures in HFS, three-way mixed ANOVA with a between-subjects factor “group” (patients with HFS and healthy controls) and two within-subjects factors “hemispheres” (left and right) and “subcortical structures” (accumbens, amygdala, caudate, hippocampus, pallidum, putamen, and thalamus) was performed on normalized volumes. *Post hoc* analysis of simple-simple effect was carried out with Bonferroni corrections using SPSS 25.0 software, and a threshold of *P* < 0.05 was considered statistically significant.

### Correlation Analyses

To examine whether subcortical structures alterations would be associated with clinical parameters, non-parametric Spearman correlation analyses were performed between volume index or vertex index and clinical parameters in patients with HFS. The significance threshold was Bonferroni corrected for multiple comparisons. Therefore, findings were considered significant if the *P* value was <0.0042 based on a *P* value <0.05/(3 subcortical abnormality indexes × 4 clinical variables per subcortical abnormality index).

## Results

### Demographics and Neuropsychological Assessment

Patients with HFS and healthy controls were matched well for age (48.80 ± 11.73 years old for patients and 49.77 ± 11.61 years old for controls, *t*_58_ = 0.32, *P* = 0.75) and sex (60% female patients vs. 60% female controls, $\chi_{1}^{2}$ < 0.01, *P* > 0.99). In addition, patients with HFS reported significant levels of anxiety (*t*_58_ = 10.06, *P* < 0.01) and felt more depressed (*t*_58_ = 9.01, *P* < 0.01) than healthy controls, which were measured by HAM-A and HAM-D, respectively. Patients with HFS had moderate spasm severity (2.93 ± 0.74) with a mean disease duration of 3.38 years. Demographic and clinical data were all presented in Table 1.

### Measures of Brain Tissue Volume

A 2 × 5 (Groups × Brain Tissue) mixed measures ANOVA was performed to examine group differences according to brain tissue volume. Notably, there was no significant Group-by-Brain Tissue interaction [*F*_(4, 9)_ = 1.218, *P* = 0.303], reflecting the fact that there were no significant group differences in brain tissue (GM, total GM, WM, CSF, and TIV) volume.

### Volume Alteration in Right Caudate

**Figure 1:**
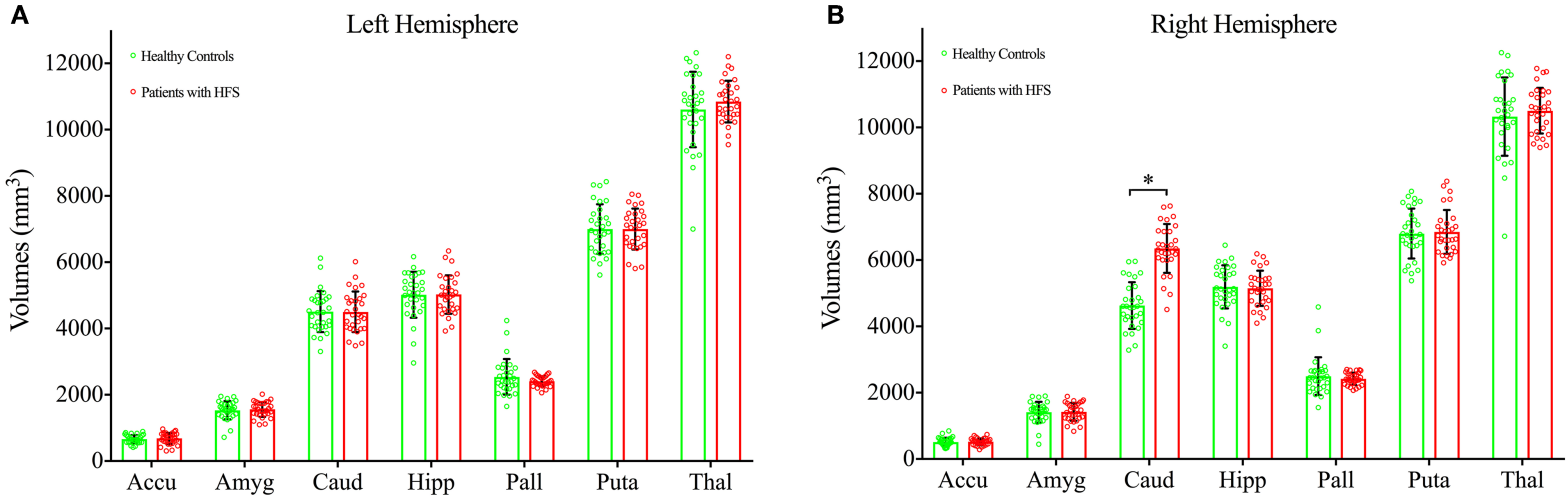
Bar graph showing the mean volumes of subcortical structures at left hemisphere **(A)** and right hemisphere **(B)** in patients with HFS (shown in red) and healthy controls (shown in green). HFS, hemifacial spasm; Error bars, mean ± standard deviation; Accu, accumbens; Amyg, amygdala; Caud, caudate; Hipp, hippocampus; Pall, pallidum; Puta, putamen; Thal, thalamus. ^*^P < 0.05.

The 2 × 2 × 7 (Groups × Hemispheres × Subcortical Structures) mixed measures ANOVA of subcortical volumes revealed a significant Groups-by-Hemispheres-by-Subcortical Structures interaction effect [*F*_(6, 812)_ = 9.13, *P* < 0.001, Table 2]. A *post hoc* analysis was then performed on the interaction effect with Bonferroni adjustment and confirmed a significant difference in right caudate with greater volume in patients with HFS compared with healthy controls [*F*_(1, 825)_ = 4.09, *P* = 0.043, Figure 1]. Other subcortical structures did not show any volume alterations in HFS patients.

**Table 2:** Normalized volumes (mm^3^) of subcortical structures in patients with HFS and healthy controls.

**Hemisphere**	**Subcortical structures**	**Patients with HFS (SD)**	**Healthy controls (SD)**
Left	Accumbens	683.47 (172.25)	660.86 (125.34)
	Amygdala	1561.99 (225.25)	1537.49 (274.04)
	Caudate	4505.34 (614.87)	4509.03 (618.91)
	Hippocampus	5027.18 (583.45)	5020.36 (690.21)
	Pallidum	2411.15 (156.19)	2545.55 (538.50)
	Putamen	7002.80 (614.24)	7002.59 (748.26)
	Thalamus	10848.63 (630.63)	10607.66 (1136.85)
Right	Accumbens	522.71 (103.95)	519.07 (127.30)
	Amygdala	1422.99 (272.76)	1413.56 (317.02)
	Caudate	6350.62 (738.88)	4632.59 (703.44)
	Hippocampus	5151.10 (535.87)	5193.64 (652.86)
	Pallidum	2418.75 (186.37)	2498.08 (568.78)
	Putamen	6854.08 (659.67)	6802.52 (752.00)
	Thalamus	10507.01 (688.90)	10332.04 (1180.14)

*HFS, hemifacial spasm; SD, Standard Deviation*.

### Shape Abnormalities in Left Pallidum and Right Caudate

**Figure 2:**
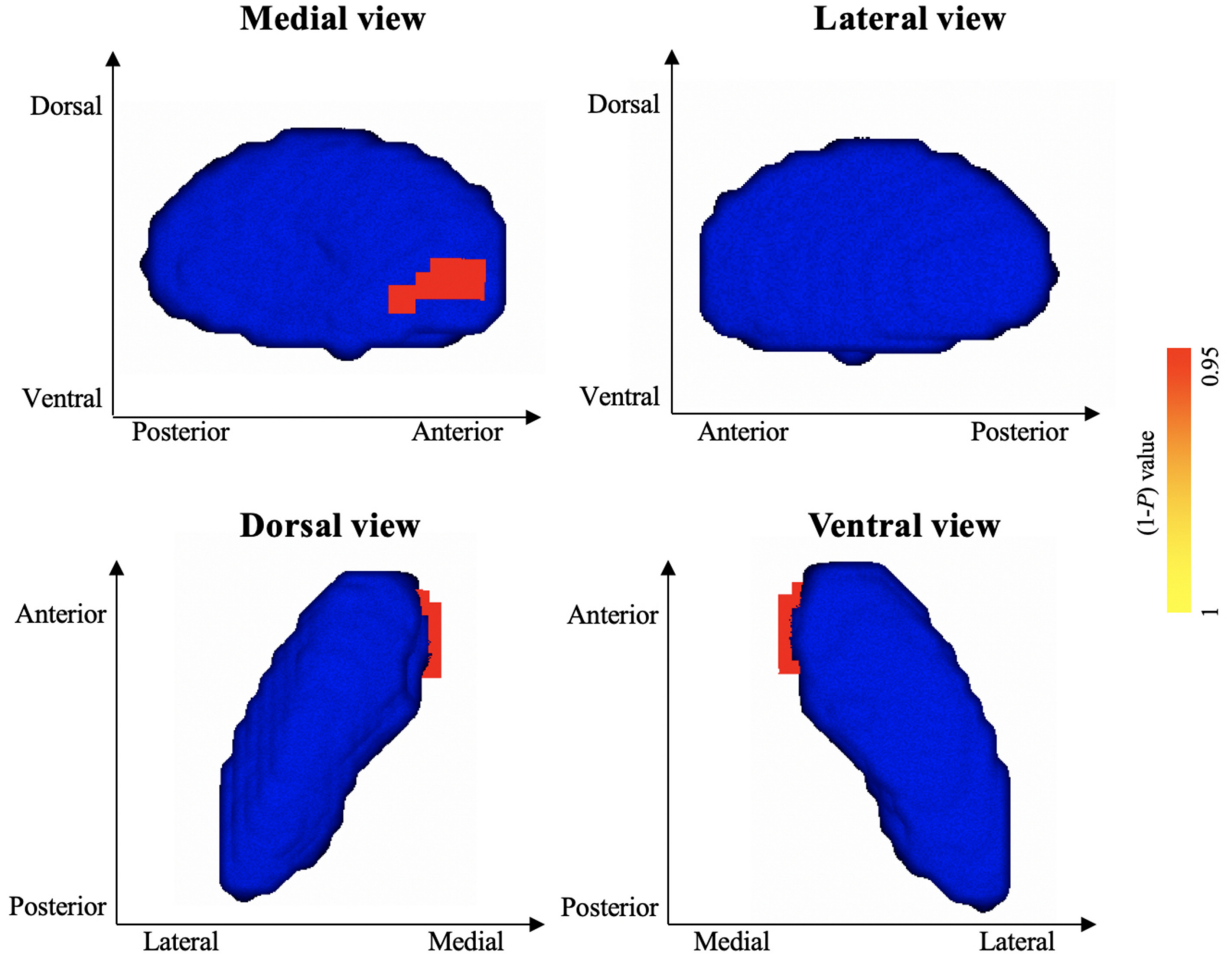
Results of vertex-wise shape analysis for left pallidum. The red-yellow colors indicate shape atrophy in anterior medial aspect of the left pallidum following patients with HFS compared to healthy controls (*P* < 0.05, FWE corrected). Blue models represent the original left pallidum structure. The color bar indicates *P* values.

**Figure 3:**
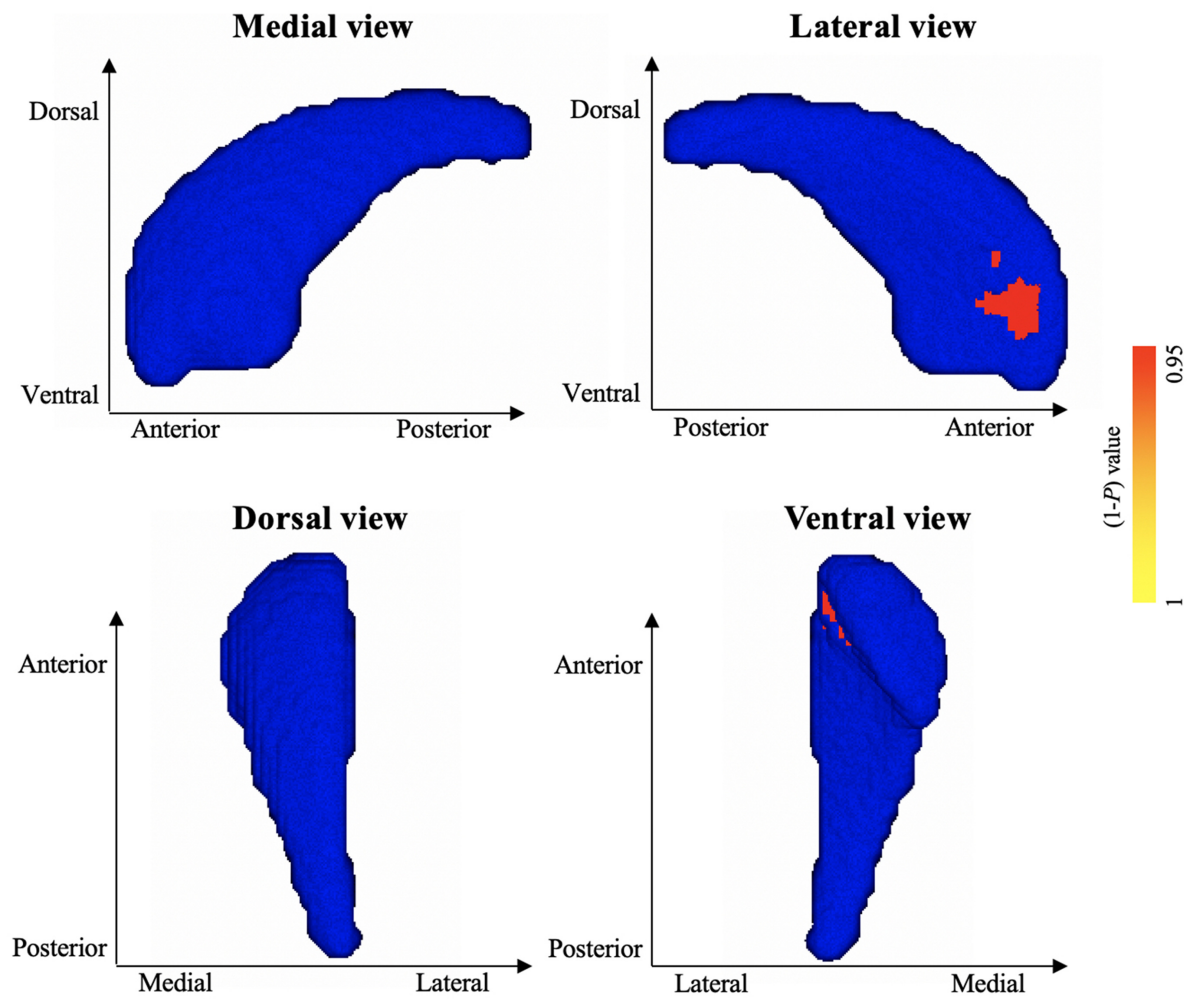
Results of vertex-wise shape analysis for right caudate. The red-yellow colors indicate shape expansion in anterior ventrolateral aspect of the right caudate head in patients with HFS compared with healthy controls (*P* < 0.05, FWE corrected). Blue models represent the original right caudate structure. The color bar indicates *P* values.

Vertex-wise shape analyses revealed areas of significant shape atrophy in the anterior medial aspect of left pallidum in patients with HFS compared to healthy controls (*P* < 0.05, FWE corrected; Figure 2 and Table 3). Besides, patients with HFS exhibited significant shape expansion in the anterior ventrolateral aspect of right caudate head in contrast to healthy controls (*P* < 0.05, FWE corrected; Figure 3 and Table 3). There were no significant alterations in other subcortical structures in patients compared to controls.

**Table 3:** Shape abnormalities in left pallidum and right caudate following patients with HFS compared to healthy controls.

**Subcortical structure**	**Side**	**Vertices**	**Peak MNI coordinates**			***t***	***P*^a^**
			**x**	**y**	**z**		
**SHAPE ATROPHY IN HFS**							
Pallidum	L	23	−11	5	−3	3.841	0.027
**SHAPE EXPANSION IN HFS**							
Caudate	R	57	20	22	−1	3.076	0.047

*MNI, Montreal Neurological Institute; HFS, hemifacial spasm; L, left; R, right*.

a *P <0.05, family-wise error corrected*.

### Subcortical Abnormalities Correlation With Clinical Parameters

**Figure 4:**
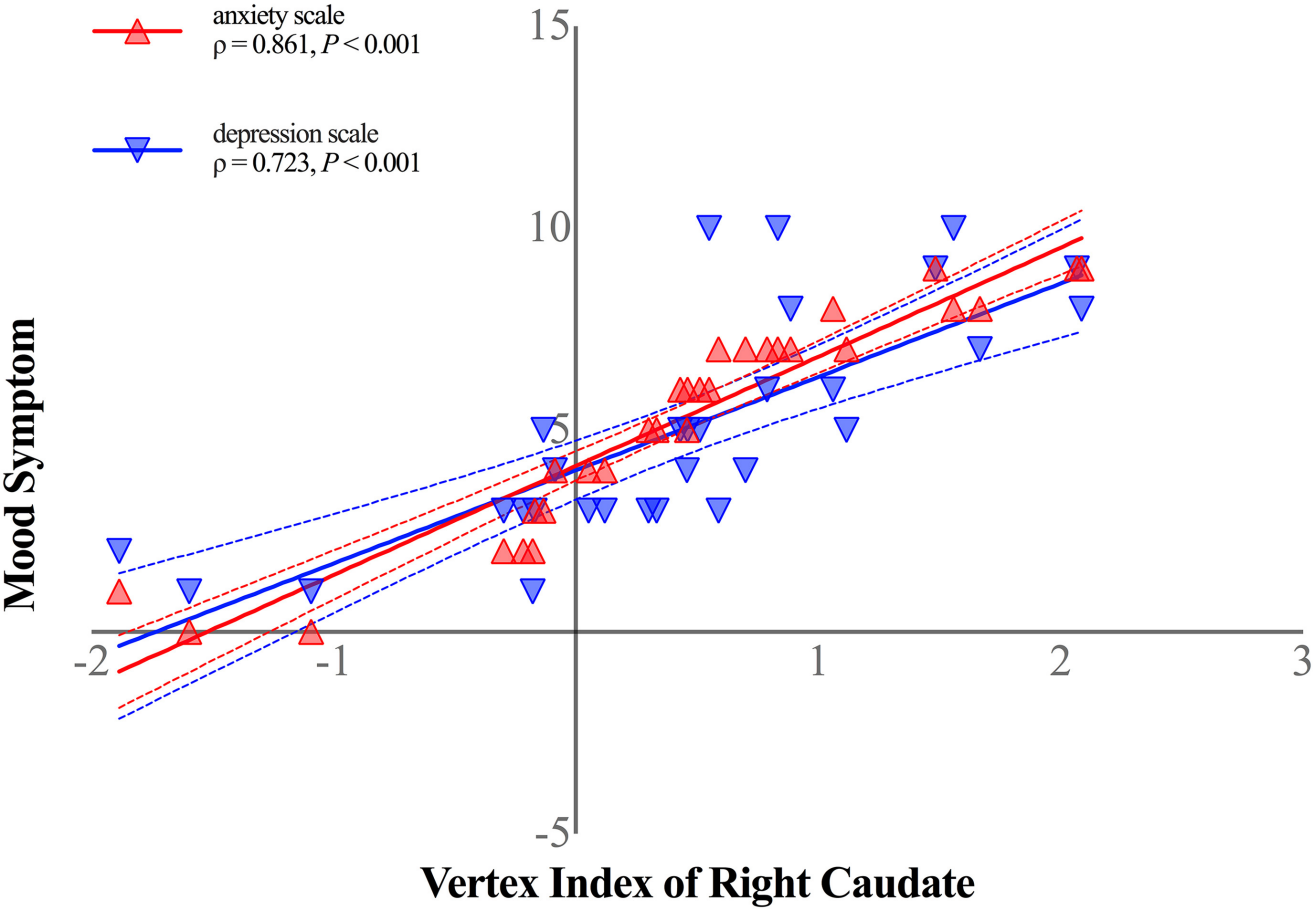
Shape alterations in right caudate was positively correlated with anxiety symptom (shown in red-filled triangle; ρ = 0.861, *P* < 0.001, Bonferroni corrected) and depression symptom (shown in blue-filled upside-down triangle; ρ = 0.723, *P* < 0.001, Bonferroni corrected) in patients with HFS. Curved dashed lines indicate 95% confidence intervals. The vertex index of anterior ventrolateral aspect of the right caudate head represented shape alterations in patients with HFS. The anxiety symptom was measured by Hamilton Anxiety Scale, and Hamilton Depression Scale was performed to assess patients' depression symptom.

Shape alterations in right caudate was positively correlated with both anxiety (ρ = 0.861, *P* < 0.001, Bonferroni corrected, Figure 4) and depression scale (ρ = 0.723, *P* < 0.001, Bonferroni corrected, Figure 4) in patients with HFS. No other significant correlations were observed in patients with HFS between other clinical parameters (disease duration, spasm severity) and subcortical structural alterations (all *P* > 0.05).

## Discussion

To our knowledge, this is the first study to employ vertex-wise shape analysis to investigate subcortical morphologic abnormalities in patients with HFS. The goal of this study was to examine subcortical aberrations in distinct subcortical regions and determine informative biomarkers of HFS. Among subcortical structures, we found increased volume specifically in the right caudate in patients with HFS compared with healthy controls. Moreover, patients with HFS exhibited significant shape atrophy in the anterior medial aspect of left pallidum, along with shape expansion in the anterior ventrolateral aspect of right caudate head when compared to healthy controls. In addition, more severe affective symptoms were associated with increased shape expansion in the anterior ventrolateral aspect of right caudate head in patients with HFS, providing support for the evidence that subcortical brain abnormalities reflect emotional disorders in patients with HFS (7).

Specifically, we observed that patients with HFS showed shape atrophy in anterior medial aspect of the left pallidum, which is known to link to motor abilities (24, 25). The pallidum is a hub inside the basal ganglia with widespread projections to all the other subcortical structures of the motor circuit (26) and receives highly convergent and topographically organized projections from the dorsal striatum (26). Pallidum alterations have been previously reported in other movement-related disorders (27, 28). To date, only one study that used VBM method reported volume alterations in the pallidum in patients with HFS (7). Possible explanations for this discrepancy may be the presence of focal disease related morphological changes within subcortical structures which does not affect the global brain volume significantly. Moreover, vertex-wise shape analyses permitted detection of shape deformations in localized areas among subcortical structures (17, 29). Shape atrophy of the left pallidum was found in HFS group, although no significant relationship was discovered between shape alteration of left pallidum and spasm severity. As the disorder progresses, spasms inevitably cause severe dysfunction of facial muscles (4). Further research is needed to determine whether pallidum abnormality is associated with other movement functions in HFS.

Furthermore, we detected increased right caudate volume following HFS. Previous studies have observed caudate volume reduction in various disorders and its associations with mood symptoms (30, 31). However, our finding of the larger-than-normal volume of caudate was inconsistent with these previous findings. This discrepancy might be owing to social phobia and depression accompanied by HFS, which may, in turn, have a cumulative effect on the caudate morphology. Moreover, patients with HFS also exhibited shape expansion in the anterior ventrolateral aspect of right caudate head. It should be noted that the ventral caudate is well-known as a vital component of the ventral striatum, and its related-neural circuits are involved in emotional processing (32, 33). Hence, shape expansion of ventral caudate in HFS may be due to the mood dysfunctions suffered by patients inducing ventral caudate alterations as this disorder progresses.

In addition, shape alteration in the right caudate was positively correlated with both anxiety and depression scale in patients with HFS, supporting an interaction that links mood dysfunctions to subcortical morphologic abnormalities in HFS. Previous studies have proved that caudate abnormality was significantly associated with depression severity in late-life depressive disorder (30) and anxiety levels in generalized anxiety disorder (34). The right caudate volume and shape abnormalities suggest that the caudate plays a critical role in mood characteristic of HFS, even though the temporal or pathophysiologic nature of caudate alterations in HFS have not been previously reported. It is unknown whether mood dysfunction leads to cellular alteration or vice versa in caudate as HFS progresses. However, because these relationships were identified in HFS, our findings suggest that caudate abnormalities could be important for further study of trans-diagnostic brain-behavior relations in HFS.

Several limitations of this study bear acknowledgment here. First, the sample size of both groups is small. Future work with larger samples is needed to confirm these findings and improve the reliability of the results. Furthermore, the affected side of HFS need to be considered with a large sample size of patients in future work. Second, we could not examine whether subcortical abnormalities observed herein precede HFS symptoms, precipitating their development, or instead represent sequelae of mood dysfunctions in our cross-sectional design. Prospective longitudinal studies are needed to delineate causes from consequences in the underlying mechanism of subcortical brain alterations in HFS.

In summary, this is the first study to employ vertex-wise shape analysis in HFS, which can reveal precisely subtle and localized alterations among subcortical structures. Compared with healthy controls, patients with HFS showed increased volume specifically in the right caudate. In addition, patients with HFS exhibited significant shape atrophy in the anterior medial aspect of left pallidum together with shape expansion in the anterior ventrolateral aspect of right caudate head. Moreover, shape alterations in right caudate were positively correlated with both anxiety and depression scale in patients with HFS. Taken together, our findings provide compelling evidence for subcortical brain abnormalities specific to HFS, and further may shed light on the pathophysiology of HFS and apply to the translational medicine.

## Data Availability Statement

The datasets generated for this study are available on request to the corresponding author.

## Ethics Statement

Written informed consent was obtained from all subjects prior to participation in accordance with the Declaration of Helsinki. This study was approved by the Ethical Committee of the first affiliated hospital of Xi'an Jiaotong University.

## Author Contributions

YW, MZ, and HX contributed to study concept/design, analysis and interpretation of data, statistical analysis, and drafting manuscript. CG and FL contributed to data acquisition, interpretation, and manuscript revision. HX and RS contributed to statistical analysis and language help.

### Conflict of Interest

The authors declare that the research was conducted in the absence of any commercial or financial relationships that could be construed as a potential conflict of interest.

## References

[1] Jannetta PJ Typical or atypical hemifacial spasm. J Neurosurg. (1998) 89:346–7. 10.3171/jns.1998.89.2.0346 9688136

[2] Colosimo C Bologna M Lamberti S Avanzino L Marinelli L Fabbrini G. A comparative study of primary and secondary hemifacial spasm. Arch Neurol Chicago. (2006) 63:441–4. 10.1001/archneur.63.3.441 16533973

[3] Pleger B. The structure of facial spasms. Clin Neurophysiol. (2016) 127:1003–4. 10.1016/j.clinph.2015.08.003 26374957

[4] Au WL Tan LC Tan AK. Hemifacial spasm in Singapore: clinical characteristics and patients' perceptions. Ann Acad Med Singapore. (2004) 33:324–8. 15175773

[5] Barker FG II Jannetta PJ Bissonette DJ Shields PT Larkins MV Jho HD. Microvascular decompression for hemifacial spasm. J Neurosurg. (1995) 82:201–10. 10.3171/jns.1995.82.2.0201 7815147

[6] Xu H Guo C Li H Gao L Zhang M Wang Y. Structural and functional amygdala abnormalities in hemifacial spasm. Front Neurol. (2019) 10:393. 10.3389/fneur.2019.00393 31114534 PMC6503044

[7] Bao F Wang Y Liu J Mao C Ma S Guo C. Structural changes in the CNS of patients with hemifacial spasm. Neuroscience. (2015) 289:56–62. 10.1016/j.neuroscience.2014.12.070 25595976

[8] Johnson MH. Subcortical face processing. Nat Rev Neurosci. (2005) 6:766–74. 10.1038/nrn1766 16276354

[9] van Schouwenburg MR den Ouden HE Cools R. The human basal ganglia modulate frontal-posterior connectivity during attention shifting. J Neurosci. (2010) 30:9910–8. 10.1523/JNEUROSCI.1111-10.2010 20660273 PMC6632831

[10] Fischi-Gomez E Vasung L Meskaldji DE Lazeyras F Borradori-Tolsa C Hagmann P. Structural brain connectivity in school-age preterm infants provides evidence for impaired networks relevant for higher order cognitive skills and social cognition. Cereb Cortex. (2015) 25:2793–805. 10.1093/cercor/bhu073 24794920

[11] Hoogman M Buitelaar JK Faraone SV Shaw P Franke B ENIGMA-ADHD working group. Subcortical brain volume differences in participants with attention deficit hyperactivity disorder in children and adults - Authors' reply. Lancet Psychiatry. (2017) 4:440–1. 10.1016/S2215-0366(17)30200-6 28495548

[12] Schmaal L Veltman DJ van Erp TG Samann PG Frodl T Jahanshad N. Subcortical brain alterations in major depressive disorder: findings from the ENIGMA Major Depressive Disorder working group. Mol Psychiatry. (2016) 21:806–12. 10.1038/mp.2015.69 26122586 PMC4879183

[13] Rimol LM Hartberg CB Nesvag R Fennema-Notestine C Hagler DJ Jr Pung CJ. Cortical thickness and subcortical volumes in schizophrenia and bipolar disorder. Biol Psychiatry. (2010) 68:41–50. 10.1016/j.biopsych.2010.03.036 20609836

[14] Opel N Redlich R Zwanzger P Grotegerd D Arolt V Heindel W. Hippocampal atrophy in major depression: a function of childhood maltreatment rather than diagnosis? Neuropsychopharmacology. (2014) 39:2723–31. 10.1038/npp.2014.145 24924799 PMC4200502

[15] Mao CP Yang HJ. Smaller amygdala volumes in patients with chronic low back pain compared with healthy control individuals. J Pain. (2015) 16:1366–76. 10.1016/j.jpain.2015.08.012 26431880

[16] Husoy AK Pintzka C Eikenes L Haberg AK Hagen K Linde M. Volume and shape of subcortical grey matter structures related to headache: a cross-sectional population-based imaging study in the Nord-Trondelag Health Study. Cephalalgia. (2019) 39:173–84. 10.1177/0333102418780632 29848110

[17] Patenaude B Smith SM Kennedy DN Jenkinson M. A Bayesian model of shape and appearance for subcortical brain segmentation. Neuroimage. (2011) 56:907–22. 10.1016/j.neuroimage.2011.02.046 21352927 PMC3417233

[18] Maier W Buller R Philipp M Heuser I. The Hamilton Anxiety Scale: reliability, validity and sensitivity to change in anxiety and depressive disorders. J Affect Disord. (1988) 14:61–8. 10.1016/0165-0327(88)90072-9 2963053

[19] Wang Y Cao D-y Remeniuk B Krimmel S Seminowicz DA Zhang M. Altered brain structure and function associated with sensory and affective components of classic trigeminal neuralgia. Pain. (2017) 158:1561–70. 10.1097/j.pain.0000000000000951 28520647

[20] Hamilton M. A rating scale for depression. J Neurol Neurosurg Psychiatry. (1960) 23:56–62. 10.1136/jnnp.23.1.56 14399272 PMC495331

[21] Cohen DA Savino PJ Stern MB Hurtig HI. Botulinum injection therapy for blepharospasm: a review and report of 75 patients. Clin Neuropharmacol. (1986) 9:415–29. 10.1097/00002826-198610000-00002 3533250

[22] Jenkinson M Beckmann CF Behrens TE Woolrich MW Smith SM. Fsl. Neuroimage. (2012) 62:782–90. 10.1016/j.neuroimage.2011.09.015 21979382

[23] Smith SM Zhang Y Jenkinson M Chen J Matthews PM Federico A. Accurate, robust, and automated longitudinal and cross-sectional brain change analysis. Neuroimage. (2002) 17:479–89. 10.1006/nimg.2002.1040 12482100

[24] DeVito JL Anderson ME. An autoradiographic study of efferent connections of the globus pallidus in *Macaca mulatta*. Exp Brain Res. (1982) 46:107–17. 10.1007/BF00238104 7067782

[25] Hardman CD Halliday GM. The external globus pallidus in patients with Parkinson's disease and progressive supranuclear palsy. Mov Disord. (1999) 14:626–33. 10.1002/1531-8257(199907)14:4<626::AID-MDS1012>3.0.CO;2-U 10435500

[26] Schwab BC van Wezel RJA van Gils SA. Sparse pallidal connections shape synchrony in a network model of the basal ganglia. Eur J Neurosci. (2017) 45:1000–12. 10.1111/ejn.13324 27350120

[27] Saini J Bagepally BS Sandhya M Pasha SA Yadav R Thennarasu K. Subcortical structures in progressive supranuclear palsy: vertex-based analysis. Eur J Neurol. (2013) 20:493–501. 10.1111/j.1468-1331.2012.03884.x 23061493

[28] Rahayel S Postuma RB Montplaisir J Bedetti C Brambati S Carrier J. Abnormal gray matter shape, thickness, and volume in the motor cortico-subcortical loop in idiopathic rapid eye movement sleep behavior disorder: association with clinical and motor features. Cereb Cortex. (2018) 28:658–71. 10.1093/cercor/bhx137 28591814

[29] Smith Y Kieval JZ. Anatomy of the dopamine system in the basal ganglia. Trends Neurosci. (2000) 23 (10 Suppl.):S28–33. 10.1016/S1471-1931(00)00023-9 11052217

[30] Butters MA Aizenstein HJ Hayashi KM Meltzer CC Seaman J Reynolds CF III. Three-dimensional surface mapping of the caudate nucleus in late-life depression. Am J Geriatr Psychiatry. (2009) 17:4–12. 10.1097/JGP.0b013e31816ff72b 18790876 PMC2970509

[31] Koo M-S Levitt JJ McCarley RW Seidman LJ Dickey CC Niznikiewicz MA. Reduction of caudate nucleus volumes in neuroleptic-naïve female subjects with schizotypal personality disorder. Biol Psychiatry. (2006) 60:40–8. 10.1016/j.biopsych.2005.09.028 16460694 PMC2768064

[32] Postuma RB Dagher A. Basal ganglia functional connectivity based on a meta-analysis of 126 positron emission tomography and functional magnetic resonance imaging publications. Cereb Cortex. (2006) 16:1508–21. 10.1093/cercor/bhj088 16373457

[33] Xu H Zhao T Luo F Zheng Y. Dissociative changes in gray matter volume following electroconvulsive therapy in major depressive disorder: a longitudinal structural magnetic resonance imaging study. Neuroradiology. (2019) 61:1297–308. 10.1007/s00234-019-02276-z 31410504

[34] Hilbert K Pine DS Muehlhan M Lueken U Steudte-Schmiedgen S Beesdo-Baum K. Gray and white matter volume abnormalities in generalized anxiety disorder by categorical and dimensional characterization. Psychiatry Res. (2015) 234:314–20. 10.1016/j.pscychresns.2015.10.009 26490569 PMC5103633

